# Characterization of a novel chicken muscle disorder through differential gene expression and pathway analysis using RNA-sequencing

**DOI:** 10.1186/s12864-015-1623-0

**Published:** 2015-05-21

**Authors:** Marie F Mutryn, Erin M Brannick, Weixuan Fu, William R Lee, Behnam Abasht

**Affiliations:** Department of Animal and Food Sciences, University of Delaware, 531 South College Ave, Newark, DE 19716 USA; Maple Leaf Farms, Leesburg, IN USA

**Keywords:** Chicken, Myodegeneration, Wooden Breast, Broiler, Myopathy, Pectoralis major, RNA-sequencing, Skeletal muscle

## Abstract

**Background:**

Improvements in poultry production within the past 50 years have led to increased muscle yield and growth rate, which may be contributing to an increased rate and development of new muscle disorders in chickens. Previously reported muscle disorders and conditions are generally associated with poor meat quality traits and have a significant negative economic impact on the poultry industry. Recently, a novel myopathy phenotype has emerged which is characterized by palpably “hard” or tough breast muscle. The objective of this study is to identify the underlying biological mechanisms that contribute to this emerging muscle disorder colloquially referred to as “Wooden Breast”, through the use of RNA-sequencing technology.

**Methods:**

We constructed cDNA libraries from five affected and six unaffected breast muscle samples from a line of commercial broiler chickens. After paired-end sequencing of samples using the Illumina Hiseq platform, we used Tophat to align the resulting sequence reads to the chicken reference genome and then used Cufflinks to find significant changes in gene transcript expression between each group. By comparing our gene list to previously published histology findings on this disorder and using Ingenuity Pathways Analysis (IPA®), we aim to develop a characteristic gene expression profile for this novel disorder through analyzing genes, gene families, and predicted biological pathways.

**Results:**

Over 1500 genes were differentially expressed between affected and unaffected birds. There was an average of approximately 98 million reads per sample, across all samples. Results from the IPA analysis suggested “Diseases and Disorders” such as connective tissue disorders, “Molecular and Cellular Functions” such as cellular assembly and organization, cellular function and maintenance, and cellular movement, “Physiological System Development and Function” such as tissue development, and embryonic development, and “Top Canonical Pathways” such as, coagulation system, axonal guidance signaling, and acute phase response signaling, are associated with the Wooden Breast disease.

**Conclusions:**

There is convincing evidence by RNA-seq analysis to support localized hypoxia, oxidative stress, increased intracellular calcium, as well as the possible presence of muscle fiber-type switching, as key features of Wooden Breast Disease, which are supported by reported microscopic lesions of the disease.

**Electronic supplementary material:**

The online version of this article (doi:10.1186/s12864-015-1623-0) contains supplementary material, which is available to authorized users.

## Background

In terms of consumption, poultry meat is highly regarded as one of the most efficient food sources; it contains high quality nutrients such as protein, and is relatively inexpensive for purchase. However, muscle disorders of commercial chickens such as deep pectoral myopathy (DPM) [1-3] and white striping [4], as well as other conditions such as pale, soft, and exudative condition (PSE) or dry, firm, and dark condition (DFD) have had an increasing negative impact on worldwide chicken meat production and quality [5]. Recently, flocks of commercial broiler chickens in the United States have developed a myopathy affecting the pectoralis major, and occasionally minor, muscle. Previously described by Sihvo et al. 2014 [6], this disease is colloquially referred to as “woody” or “wooden” breast, due to clinical and microscopic changes to the muscle resulting overall in palpable severe hardness of the breast muscle. Hereafter this syndrome will be referred to as Wooden Breast. The superficial area of the pectoralis muscle tends to be more affected than deeper portions of the muscle. Lesions can be detected clinically through manual palpation of the breast muscle in live birds as early as 3 weeks in age. Owens (2014) [7] has recently reported that severe cases of Wooden Breast disease can likely affect about 10% of an entire flock; but it was also noted that some degree of Wooden Breast disease has been anecdotally reported to affect up to 50% of a flock. The disease is emerging on a global scale, already present in Finland [6] and to the authors’ knowledge in several other countries.

Sihvo et al. 2014 [6] specifically studied the microscopic and histologic properties of the pectoralis muscle from chickens affected with Wooden Breast. Although histological evidence indicated multifocal degeneration and necrosis of muscle tissue with infiltration of inflammatory cells, the underlying etiology of this disorder was not apparent [6]. Wooden Breast has also previously been reported in the United States by Bilgili 2013 [8] at the University of Auburn in Alabama with the general phenotypic properties of the myopathy described being in agreement with Sihvo et al. 2014 [6]. Lesions associated with the myopathy appear to be aseptic, superficially-located, and include muscle fiber fragmentation, hyalinization, and swelling with replacement by fibrous connective tissue, as well as an influx of macrophages and other immune cells and the occurrence of irregular adipose tissue throughout the muscle. Bilgili 2013 [8] hypothesizes that these features may be associated with localized hypoxia due to a reduction in capillary supply.

Previously described muscle disorders in chickens, such as white striping and nutritional myopathy, have been shown to affect the integrity of the pectoralis major muscle in chickens. White striping is identified through the observation of white fatty lines on the breast muscle running in the same direction as the muscle fibers of the breast muscle [9]. It is speculated that white striping is caused from a mineralization of fat on myofibers, along with general necrosis of the muscle [9]. Interestingly, there may be a correlation between white striping and Wooden Breast, as birds affected by Wooden Breast are likely to show gross features of white striping [6]. It is also possible that the two disorders represent a disease spectrum, with white striping cases being the less severe form of the myopathy as compared to Wooden Breast.

Nutritional myopathy also affects the breast muscle of broiler chickens and is generally associated with low levels of vitamin E and selenium in diet [10]. The main clinical features of nutritional myopathy can be observed grossly as white striations in the skeletal muscle [11] and histologically as degradation, necrosis, mineralization, and regeneration of myofibers in the muscle [10]. Although some of the characteristics of white striping and nutritional myopathy overlap with lesions of Wooden Breast, the grossly palpable hardness of the pectoralis muscle remains unique to Wooden Breast. Additionally, unlike in nutritional myopathy, no lesions are observed in other muscle groups, such as the gizzard or heart, beyond the pectoralis major and minor in Wooden Breast.

The overall objective of this paper is to use RNA-sequencing to identify biological and molecular pathways that are involved in the underlying etiology and pathogenesis of this emerging muscle disorder of the pectoralis muscle in commercial broiler chickens. We used RNA-sequencing as this powerful technology provides comprehensive and accurate gene expression data, performing better than previous technologies such as microarrays [12]. Also, useful tools have been generated for RNA-sequencing data analyses that make for user-friendly and efficient analysis of the data.

## Methods

Chickens used in this study were all males from a high breast meat yield purebred line of broiler breeder chickens raised on the floor, in pens of 300, by Heritage Breeders in the Delmarva region of the United States. Birds were given free access to both feed and water, and were housed according to optimal industry growing standards. Birds were euthanized at 47 days of age by cervical dislocation. After euthanasia, pectoral muscle samples were collected at necropsy. Based upon clinical examination and manual palpation of pectoral muscle for evidence of palpable “hardening", eight affected and eight unaffected birds were identified; affected birds were those deemed to have “severe” to “moderately-severe” lesions, while unaffected birds showed no apparent gross lesions. Samples from the pectoralis major were taken from the caudal aspect of the right superficial pectoral muscle, perpendicular to myofibers and to the keel bone. Samples were taken with the Keyes biopsy punch (8 mm) manufactured by HNM. Roughly one to two grams of tissue was extracted from each sample location and immediately flash frozen in liquid nitrogen. The protocols were submitted to, and the use of the collected samples for research was approved by, the University of Delaware Agricultural Animal Care and Use Committee (protocol number: 44 12-15-13R).

Total RNA was extracted from the breast muscle tissue samples using the *mir*Vana™ miRNA Isolation Kit according to the manufacturer’s protocol (Life Technologies®). Multiple quality checks were performed to assess the RNA quality and integrity using NanoDrop 1000 and Angilent Bioanalyzer 2100 to measure RNA concentrations following the established manufacturer protocol. The RNA integrity number of samples was larger than 8, which is considered acceptable for cDNA library preparation.

Eleven samples, five affected and six unaffected, were prepared to create cDNA libraries for RNA-sequencing. All samples used for RNA-sequencing were also strictly verified for correct identity (Additional file 1). cDNA libraries were constructed following the TruSeq Stranded mRNA Sample Prep Kit following the protocol for low sample (LS). After construction, the 11 cDNA libraries were normalized as suggested by the manufacturer, to 10 nm/μl using Tris buffer (Tris-Cl 10 mM, 0.1 % Tween 20, pH 8.5). The 11 uniquely indexed, normalized libraries were pooled together in 2 tubes and submitted to the Delaware Biotechnology Institute (DBI) for 101 nucleotides paired-end sequencing on 2 lanes of a flow cell using the Illumina Hiseq 2000 system and on 1 lane of a flow cell using the Illumina Hiseq 2500 system.

The quality of the data received from DBI was checked with FastQC v0.10.1 [13]. RNA sequencing reads that passed this quality control measure were then mapped to the chicken reference genome (Galgal4.0 Nov 2011) using TopHat v2.0.4 [14]. Parameters of TopHat were set to only report read alignments if 1) both reads in a pair could be mapped, 2) there were no more than two mismatches per read, 3) concordant mapping occurred for both reads in a pair, 4) no more than one mismatch per segment (−−segment-mismatches), and 5) mean inner distance between mate pairs set at 110. TopHat cut up input reads into smaller segments and mapped these segments independently. The default parameter (25 nucleotides) was used for segment length.

Cufflinks v2.1.1 [15] was used for the differential expression analysis: parameters of Cuffdiff were set to allow normalization of the number of fragments mapping to individual loci to improve the strength of differential expression analysis (change in default-- added –N/--upper-quartile-norm parameter). Cuffdiff labeled genes as significant or not significant based on whether the p-value of statistical test for differential expression was greater than the false discovery rate (FDR) after Benjamini-Hochberg correction for multiple testing [15]. Genes with a q-value or FDR-adjusted p-value of < 0.05 and a fold change greater than 1.3 were considered statistically significant and submitted to Ingenuity Pathway Analysis (IPA®) [16] for functional annotation and identification of significant biological pathways and upstream regulatory factors. The fold change for each gene was provided by Cuffdiff as log_2_(fold change) and was then calculated as 2 ^log^_2_^(fold change)^ for genes with a positive log_2_(fold change) and −1/(2 ^log^_2_^(fold change)^) for genes with a negative log_2_(fold change) before submission to IPA.

To verify the gene expression data we used NanoString nCounter® technology previously described by Geiss et al. 2008 [17]. We selected 192 target genes, along with 12 housekeeping genes across various RNA-sequencing projects within our lab to be measured. The 11 samples used in this paper were sent to NanoString Inc. (Seattle, WA, USA), where they custom designed 204 individual probes for target sequences. NanoString Inc. hybridized 100 ng of each RNA sample to the 204 designed probes. After incubation, the samples went through the nCounter® Prep Station and the Digital Analyzer for final transcript counting. Control factors and housekeeping genes were used to normalize the data for further analysis [17]. Finally, the log_2_(fold change) for gene expression between affected and unaffected birds were calculated from the NanoString results to be compared with the log_2_(fold change) of gene expression from the RNA-sequencing results.

## Results and analysis

The total number of reads per sample provided from RNA-sequencing can be found in the Additional file 2. The average number of reads across all 11 samples was 97,890,272.

**Fig. 1:**
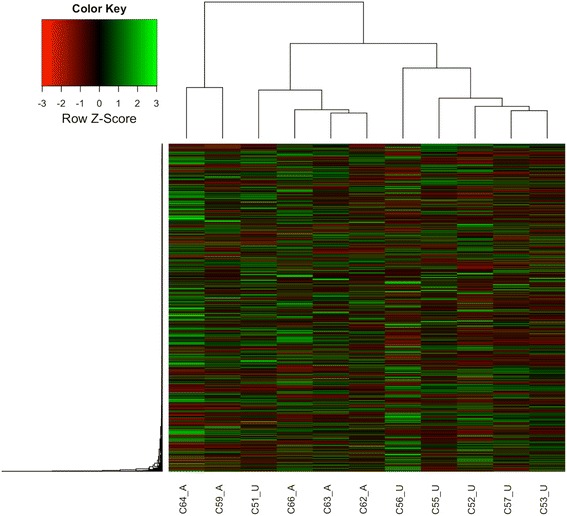
Heatmap gene cluster classification for Wooden Breast and unaffected muscle samples. Using the expression for each gene (in rows) and sample (in columns), the heatmap is generated by the R package “plots”. The expression levels of each gene across samples are shown as Z-scores scaled by their FPKM from RNA-seq. The scaled expression values are color-coded according to the legend. The dendrogram depicting hierarchical clustering is based on the expression of all genes

Expression data for each gene within each sample was used to create a heatmap for cluster classification. The heatmap was generated using the R package “plots” (Fig. 1). This cluster analysis revealed a clear separation between affected and unaffected samples. It should be noted that one sample (Sample 51 unaffected) was seemingly clustered in the incorrect group. We believe that this inconsistency is likely due to the broad range of disease severity possible in Wooden Breast. It is reasonable to suspect that the syndrome was in fact developing in Sample 51, but not yet expressed in terms of the palpable muscle firmness. This suggests clinicopathological heterogeneity in the classification of this disease.

We verified our RNA-sequencing data using the Nanostring nCounter gene expression assay. Comparison of the log_2_(fold change) for normalized NanoString count data against our RNA-sequencing data using the Pearson pair-wise correlation coefficient were very high, with a value of 0.861 across all overlapped genes tested in RNA-seq.

Ensembl ID of differentially-expressed (DE) genes, i.e., genes with a q-value < 0.05 and a fold change higher than 1.3, were submitted to IPA® for functional annotation as well as mapping to canonical pathways and identifying upstream regulators. The differentially expressed genes with positive or negative fold change values are considered to be, respectively, upregulated or downregulated genes in affected birds relative to their expression in unaffected birds. Among the 1637 significant DE genes identified, IPA recognized 1390 of these genes for analysis. The Ingenuity Knowledge Base®, which supports the mapping of genes, relies on ortholog information for only human, mouse, and rat. Some genes went unmapped because there is no known ortholog with either of these species (human, mouse, rat). A summary of the top ten up-regulated and top ten down-regulated genes in the affected birds, along with the fold change for each gene is presented in Table 1.

**Table 1:** Top ten upregulated and downregulated genes with fold change for Wooden Breast myopathy-affected birds

Gene	Description	RNA-seq fold change	Nanostring fold change
ENSGAL00000009603	Prolactin-like protein precursor	43.9	N/A
CA3	Carbonic anhydrase III	28.5	25.5
CLEC3A	C-type lectin domain family 3, A	25.3	N/A
KPNA7	Karyopherin alpha 7	24.4	8.0
CSRP3	Cysteine and glycine-rich protein 3	23.2	27.3
CRISP1	Cysteine-rich secretory protein 1	21.5	N/A
FAM132A	Family sequence similarity 132, A	18.0	N/A
MB	Myoglobin	14.3	12.4
CRH	Corticotropin releasing hormone	14.3	13.3
PHGDH	Phosphoglycerate dehydrogenase	11.6	5.4
GC	Group Specific Component	−48.0	N/A
PIT54	PIT 54 protein precursor	−46.0	N/A
FGA	Fibrinogen alpha chain	−20.8	−3.0
FGG	Fibrinogen gamma chain	−18.1	−2.8
OGCHI	Orosomucoid 1	−16.9	N/A
ALB	Albumin	−16.8	−4.0
ENSGAL00000016859	Uncharacterized protein	−14.9	N/A
FGB	Fibrinogen beta chain	−13.3	−3.8
APOH	Apolipoprotein H	−13.1	N/A
YFV	MHC I antigen precursor	−12.9	N/A

This table provides a summary of both the top ten upregulated and downregulated genes found among affected chickens within the sample population. Positive and negative fold change values support, respectively, upregulation and downregulation of gene expression in affected birds relative to the gene expression in unaffected birds. The fold change for each gene is also presented along with the fold change for each gene that was tested using the NanoString nCounter® Technology to verify our expression data

A summary of the genes identified in the IPA® category of “Top Biological Functions” is presented in Table 2 and contains the subcategories of “Diseases and Disorders”, “Molecular and Cellular Functions”, “Physiological System Development and Function” and “Top Canonical Pathways”. Also presented in Table 2 is the significance or P-value listed for each subcategory. IPA determines the P-value for each subcategory by dividing the number of DE genes (# of Genes) for each subcategory by the total number of known genes within that specific subcategory present in the IPA software database “Query Ingenuity Knowledge Base” (IPA website). The P-value for the top canonical pathways is also determined in the same manner. The “Genes” and “Ratio” columns in this subcategory were determined by dividing the number of observed DE genes by the total number of genes within that specific canonical pathway present within the IPA software database: “Query Ingenuity Knowledge Base” [16].

**Table 2:** Top biological functions of genes differentially expressed between Wooden Breast myopathy-affected and unaffected birds as presented by Ingenuity Pathway Analysis (IPA®)

Biological Functions	*P-value*		# Molecules
Diseases and disorders:			
Cancer	3.18E-31 – 4.08E-05		1208
Gastrointestinal Disease	9.84E-26 – 3.06E-05		890
Cardiovascular Disease	7.45E-21 – 4.06E-05		293
Hepatic System Disease	1.86E-20 – 3.06E-05		593
Neurological Disease	1.95E-17 – 3.92E-05		407
Molecular and Cellular Functions:			
Cellular Assembly and Organization	7.25E-19 – 1.14E-05		321
Cellular Function and Maintenance	7.25E-19 – 2.06E-05		402
Cellular Movement	1.80E-17 – 3.43E-05		322
Cell Morphology	1.51E-16 – 2.53E-05		387
Cellular Growth and Proliferation	2.81E-16 – 3.82E-05		476
Physiological System Development and Function:			
Nervous System Development/Function	9.74E-14 – 3.90E-05		195
Tissue Development	9.74E-14 – 3.82E-05		501
Cardiovascular System Dev./Function	2.75E-13 – 3.70E-05		237
Organismal Development	5.44E-13 – 3.63E-05		432
Embryonic Development	9.51E-13 – 3.10E-05		306
Top Canonical Pathways:	P-Value	Genes	Ratio
Hepatic Fibrosis/Stellate Cell Activation	2.83E-12	43/197	0.218
Epithelial Adherens Junction Signaling	2.39E-08	30/146	0.205
Actin Cytoskeleton Signaling	3.18E-07	36/217	0.166
Axonal Guidance Signaling	1.27E-06	56/433	0.129
Coagulation System	1.39E-06	12/35	0.343

Top Biological Functions of DE genes associated with affected chickens from the sample population within this study provided by Ingenuity Pathway Analysis (IPA®). The P-value provided by this table was determined by IPA by comparing the number of genes found within each category (# of Genes) with the number of known genes within the IPA software system. The P-value for the “Top Canonical Pathways” is also determined by IPA in the same manner

The resultant RNA-seq profile for Wooden Breast indicated by these analyses includes evidence of altered intracellular calcium, hypoxia, oxidative stress, potential fiber-type switching, and cellular repair within affected muscle. Additionally, there is direct correlation between RNA-sequencing data and the histologic lesions observed in the Wooden Breast myopathy.

## Discussion

### Intracellular buildup of calcium

Through analysis of DE genes in this study, there is ample evidence to suggest that intracellular calcium overload with the potential to impact membrane integrity is occurring in the pectoralis major muscle of broilers affected with Wooden Breast. The ATPase, Ca++ transporting, cardiac muscle, slow twitch 2 (*ATP2A2)* gene, which is upregulated in birds affected with Wooden Breast, encodes for sarco(endo)-plasmic reticulum Ca^2+^ -ATPase isoform 2a and 2b (SERCA2a/SERCA2b) [18]. *SERCA2a* and *SERCA2b* differ in the location where they are generally expressed with *SERCA2a* showing high levels of expression in the heart and in slow-twitch skeletal and smooth muscle, whereas *SERCA2b* is expressed in all tissues [18]. SERCA2 is a critical component of the endoplasmic reticulum (ER) and sarcoplasmic reticulum (SR) and acts as a pump to sequester [19] and translocate Ca^2+^[20]. SERCA pumps also have a role in contraction and relaxation of myofibrils, maintaining calcium homeostasis by sustaining the correct calcium levels required for relaxation and as well as maintaining “reloading” calcium levels needed for contraction [18, 20]. Increased expression of *SERCA2* leads to quicker calcium uptake and larger amounts of calcium loading within the ER [21]. Previously, a study in chickens showed that *SERCA* expression could be influenced by extrinsic factors such as disease [22]. The upregulation of *ATP2A2* in Wooden Breast birds may be occurring in response to increased amounts of intracellular calcium within muscle cells, potentially leading to up-take and loading calcium within the sarcoplasmic reticulum at faster than normal rates in affected birds.

Parvalbumin (*PVALB*), another upregulated gene in Wooden Breast birds, is essential to calcium buffering and regulating calcium concentrations within the cell [20]. PVALB acts to insure relaxation of the muscle through rapidly sequestering calcium from the sarcoplasm of the cell [20]. The upregulation of *PVALB* in birds affected within the current study may be compensatory to avoid a significant rise in intracellular calcium levels. This type of compensatory change has also been hypothesized in other muscle disorders such as Duchenne dystrophy in humans, in which excess calcium negatively impacts the muscle [23]. Therefore, it seems that increases in *PVALB* expression may act to stop hypercontraction of the muscle by inducing relaxation through calcium buffering and binding.

Abnormal accumulation of intracellular calcium can occur because of damaged cellular membranes of muscle cells or during episodes of metabolic imbalance between calcium and other ions [24]. The resultant damage to muscle cells can occur in various ways; it is hypothesized that extra calcium may activate proteases or lipases within the cell, which eventually leads to muscle fiber breakdown and an increase in calcium influx, initiating a “vicious cycle” of further muscle damage and calcium release [24]. It is also thought that excess calcium can impede mitochondrial performance ultimately decreasing the available energy supply when calcium cannot be effectively pumped out of the cell [24, 25]. Calcium overload can result in the activation of calpains and phospholipase A_2_ (PLA_2_), increase the overall production of ROS, and may also lead to excess mitochondrial calcium [25]. Damage to the sarcolemmal membrane occurs after activated PLA_2_ disrupts mitochondrial function causing the formation of lysophospholipids [25] that in turn disrupt the sarcolemmal membrane [26]. In the current study, 2 membrane-associated phospholipase genes, the *PLA2G4A* gene, encoding cytosolic phospholipase A_2_ (PLA2), and phospholipase B1 (*PLB1*) were upregulated in birds affected with Wooden Breast. *PLA2G4A* has previously been implicated in broad spectrum skeletal muscle myopathies in broiler chickens [27]. Overall, the upregulation of *PLA2G4A* and *PLB1*, and increased intercellular calcium may be causing changes in membrane integrity in Wooden Breast chickens.

### Hypoxia

A number of significant differentially expressed genes in the present study have been found to be regulated by HIF-1 (Hypoxia-inducible factor- 1) (HIF-dependent expression), suggesting a hypoxic state in Wooden Breast. These genes include procollagen-lysine, 2-oxoglutarate 5-dioxygenase 2 (*PLOD2*), transforming growth factor beta 3 (*TGFB3*), asparagine synthetase (*ASNS*), matrix metallopeptidase 2 (*MMP2*), transient receptor potential cation channel, subfamily A, member 1 (*TRPA1*), muscle-specific carbonic anhydrase III (*CA3*), and 6-phosphofructo-2-kinase (*PFKFB3*). Hypoxia-inducible factor 1 (HIF-1) is an important transcription factor to consider when discussing hypoxia. HIF-1 regulates many genes involved in angiogenesis, energy metabolism, vasomotor regulation, and cell proliferation as well as cell survival, and it is critical in maintaining oxygen homeostasis within the cell [28-30]. Although genes encoding the two subunits of HIF-1, i.e., hypoxia-inducible factor 1-alpha (*HIF-1A*) and aryl hydrocarbon receptor nuclear translocator (*ARNT*), are not differentially expressed between normal birds and those affected by Wooden Breast, our significant gene list suggests the activation of this transcription factor in affected birds. It has been previously reported that in skeletal muscle *HIF-1A* is highly expressed in both hypoxic and normoxic conditions [31], which may give insight into why *HIF-1A* is not differentially expressed between affected and unaffected birds in the present study, though there is specific evidence for hypoxic conditions in the Wooden Breast profile.

For example, *PLOD2*, which is upregulated in wooden breast-affected birds, is an important gene associated with extracellular matrix composition, specifically extracellular matrix stiffening and collagen alignment, such as with fibrosis [32]. *PLOD2* expression is essential for correct collagen formation in response to hypoxic events [33]. HIF-1 increases *PLOD2* expression during hypoxia, which increases collagen content and impacts the extracellular matrix by increasing cell adhesion, elongation, and motility, creating a stiff cellular environment [33]. The upregulation of *PLOD2* in affected birds correlates with the histological characterization of fibrosis previously reported in chickens with Wooden Breast [6] and observed histologically in our laboratory (data not shown). Furthermore, this gene may play an integral role in the accompanying stiffness of Wooden Breast birds, possibly related to changes within the extracellular matrix surrounding muscle cells in the breast muscle.

*TRPA1* is another HIF-dependent gene upregulated in affected birds in our study. Through the formation of calcium and zinc ion channels, *TRPA1* acts as a sensor to reactive compounds, such as oxidative and thiol compounds, which are mainly produced during oxidative stress [34]. *TRPA1* also mediates inflammation [35]. Hatano et al. 2012 [36] recently provided evidence for the upregulation of *TRPA1* by HIF. Their findings included the induced expression of *TRPA1* by TNF-alpha and IL1-alpha are primarily regulated through HIF. Although *TNF-alpha* is absent in chickens [37, 38], many *TNF* family members are differentially expressed between unaffected and affected birds in our study. Also, inflammation is a key characteristic of the breast muscle in Wooden Breast birds, which is critical as inflammatory cytokines and mediators induce *TRPA1* expression [36]. It should also be noted that within the current study, additional inflammatory cytokines IL-15, and IL-18 are both upregulated in affected birds.

Matrix Metallopeptidase-2 (*MMP2*), characterized as a HIF-dependent gene, is also upregulated in birds affected with Wooden Breast. MMP2 is a member of a large family of matrix metalloproteases and is typically associated with the degradation of cellular components such as the basement membrane and type IV collagen [39]. MMP2 also plays a role in regulating vascularization and response to inflammation [40]. Milkiewicz & Haas 2005 [41] observed that during instances of mechanical stretch to muscle, both the expression of *HIF-1A* and *MMP2* increased. More specifically, they detected increased gene expression of *MMP2* in capillary endothelium of muscle cells. Although the likely activation of HIF-1 within the current study may be the primary cause for the upregulation of HIF-dependent *MMP2*, it must also be considered that *MMP2* expression may be increased due to vascular smooth muscle stretch and remodeling secondary to venous damage or inflammation in birds affected with Wooden Breast.

Other differentially expressed genes within our study such as transforming growth factor, beta 3 (*TGFB3*), asparagine synthetase (*ASNS*), and muscle specific carbonic anhydrase III (*CA3*) were previously reported as regulated by HIF-1. *TGFB3*, which is upregulated in affected birds in our study, has been previously found to be upregulated in traumatized muscle during instances of chronic inflammation and regenerating muscle tissue, which is evident microscopically in this study (data not shown), and which stimulates a hypoxic microenvironment [42]. In the current study, *ASNS* is upregulated in affected chickens. Cui et al. 2007 [43] detail the impact that both hypoxic conditions and insufficient glucose levels have on *ASNS* in relation to tumor growth. They reported a significant increase in expression of *ASNS* in response to inadequate glucose levels. The upregulation of *ASNS*, possibly due to low glucose levels, also correlates with previous observations, such as the downregulation of *PFKFB3*, suggesting deficient glucose metabolism. *CA3* is also differentially expressed between the two groups of chickens in the present study, and is upregulated in affected birds. CA3 has previously been found to increase within the muscle tissue of human hypoxia-trained athletes [44]. It is suggested that the observed increase in CA3 may be a mechanism to increase the rate of H^+^ ions delivery to the interstitial fluid of the cell in order to combat muscle fatigue [44]. However, as discussed under the “oxidative stress” section, the upregulation of *CA3* in affected muscle may be primarily associated with the antioxidant role of its protein product [45].

*PFKFB3*, a HIF-dependent gene, is found to be downregulated in birds affected with Wooden Breast in the current study. This is contrary to previous work, which demonstrated that under hypoxic conditions *PFKFB3* expression typically increases [46]. During hypoxic conditions, cells may switch from oxidative phosphorylation to glycolysis in order to maintain energetic homeostasis [46]; PFKFB3 plays a major role in this process as an activator and regulator of glycolysis and glucose consumption [47]. Since *PFKFB3* is downregulated in our study, it may be possible that glucose metabolism is regulated via a different mechanism under the hypoxic conditions associated with Wooden Breast in chickens. Further explanation for this finding remains to be studied.

Additionally, hypoxic conditions tend to stimulate satellite cell proliferation [31], which is extremely important for skeletal muscle regeneration [48], highlighting the significant role of oxygen concentrations and oxygen as a signaling molecule regulating satellite cells [49]. Hypoxic conditions can also influence the phenotype and differentiation ability of satellite cells [31]. Satellite cells constitute a supply of nuclei for muscle fibers during muscle growth and significantly contribute to compensatory muscle hypertrophy. Muscle growth in chickens, after hatch, can also largely be attributed to hypertrophy [50]. In broilers, satellite cells have high proliferation and differentiation rates just after hatch [51]. After this time, however, proliferation rates tend to drop off [52], but satellite cells continue to contribute to myofiber hypertrophy [51]. Myogenesis, or muscle formation, is regulated in part by the transcription factor paired-box 3 (PAX3), which is upregulated in birds affected with Wooden Breast in the present study. It has been suggested that PAX3 activates genes associated with myogenic regulatory factors and after muscle injury, *PAX3* expression increases within cells that express myogenic regulatory factors [53]. Overall, the stimulation of satellite cell expansion under hypoxic conditions has the ability to lead to muscle hypertrophy during myogenesis and myoregeneration, which has been observed microscopically in Wooden Breast muscle tissues in diagnostic cases analyzed in our laboratory (data not shown). Since all of the birds collected in this study were at the same developmental stage, it is likely that the upregulation of *Pax3* in affected birds is due to myoregeneration following muscle damage, rather than due to generalized muscle growth, though it is possible that this expression difference could also be linked with excessive muscle growth, characteristic of birds with Wooden Breast.

### Oxidative stress

Oxidative stress caused by an increase in reactive oxygen species (ROS), may be a major contributor to the Wooden Breast phenotype. Many DE genes between the two groups of broilers being studied suggest an increase in ROS in birds affected with Wooden Breast. Increases in ROS within the skeletal muscle are typically generated as a response to increased contractile activity or after periods of major muscle disuse [54]. Increases in ROS can also occur following mitochondrial dysfunction as a result of oxidative phosphorylation [55]. A major increase in ROS is detrimental to muscle cells due to cytotoxicity and can impact cells by damaging or altering proteins and membrane lipids, thus altering cellular integrity [54]. It is also known that increased concentrations of ROS may impact calcium myofibril sensitivity and calcium release from the sarcoplasmic reticulum, resulting in damage to the contractile ability of muscle cells specifically [56]. Furthermore, increases in ROS also have the ability to impact and regulate cellular signaling pathways that are critical to gene expression and ultimately act on skeletal muscle remodeling and adaptation [54].

Oxidative stress can elicit high amounts of stress within the body and to specific tissues that undergo direct increases in ROS. Because of oxidative stress, the activation of stress response-related pathways and genes are appropriate reactions to prevent further damage. In the current study, the differential expression of many genes within the heat shock family and other stress response-related genes suggests the occurrence of oxidative stress within the breast muscle of affected chickens. The genes within the heat shock family that are upregulated in affected chickens include: heat shock transcription factor 2 (*HSF2*), heat shock 27 kDa protein family, member 7 (*HSPB7*), heat shock 70 kDa protein 4-like protein (*HSPA4L*), and heat shock 105 kDa/110 kDa protein 1 (*HSPH1*). Another gene within this family that is also differentially expressed but found to be downregulated in affected chickens is FK506 binding protein (*FKBP51*). Heat shock proteins are broadly regulated, and are a fairly large gene family, meaning that members respond to different stimuli [57]. This may explain why some heat shock protein family members in our study are upregulated while simultaneously another is downregulated. Heat shock proteins serve many functions such as molecular chaperones, guiding protein folding, and preventing protein buildup. During stressful events such as oxidative stress, the expression of heat shock proteins is known to increase greatly [58]. On the whole, the heat shock proteins are activated in order to stimulate a pro-survival response during oxidative damage [59]. Therefore, the upregulation of most heat shock proteins in affected birds, indicates the possibility of oxidative stress damages within muscle cells, and also confirms the likelihood of cellular stress in birds with Wooden Breast. In addition, corticotrophin-releasing hormone (*CRH*), a major factor important to stress response pathways, was identified as a top upregulated gene in affected birds (fold change = 14.31) in the current study. *CRH* expression has been previously found to aid in the protection of neurons against oxidative stress [60]. CRH is typically expressed in tissues outside the brain during inflammatory events [61]. The accumulation of immune cells in Wooden Breast birds may be a potential source of CRH, as some immune cells are known to secrete CRH [61].

Other well-known myopathies, such as Vitamin E/Selenium deficiency-related (nutritional) myopathy, render skeletal muscle to be more susceptible to oxidative damages by depleting or overwhelming the antioxidant defense system [10, 62]. Such nutritional myopathies show overlapping microscopic lesions to Wooden Breast [6], such as muscle cell death, degradation and regeneration of myofibers [10], and white striations (adipose) within the musculature [11], vascular damage, and fibrosis [63]. These myopathies are frequently found in agricultural species, such as poultry, and negatively impact meat quality [10, 11, 64]. Most research surrounding nutritional myopathies, such as studies mentioned above, focus strongly on diet supplementation and feed intake [10, 62]. However, we can likely rule out nutritional deficiency as a major factor in Wooden Breast development because both affected and unaffected birds are exposed to the same diet. Additionally, unlike in nutritional myopathies, Wooden Breast appears to only affect the pectoral muscle rather than multiple muscles, gizzard, and heart, and myofiber mineralization is minimal to absent.

Nevertheless, selenoprotein-related deficiencies are strongly associated with oxidative stress damage, as selenoproteins are essential for counteracting oxidative stress [65]; the selenium-protein complex includes oxidative stress regulatory proteins such as glutathione peroxidase and thioredoxin reductase [65]. Within this study, selenoprotein O (*SELO*) is found to be downregulated in Wooden Breast affected broilers. SELO was recently characterized by Dudkiewicz et al. 2012 [66], and it is suggested that it has important roles in oxidative stress response and may be a factor in molecular transport and efflux systems [66]. Interestingly, gene expression for chickens with dietary selenium deficiency (i.e. nutritional myopathy or White Muscle Disease) shows a downregulation of *SELO*, along with other selenoprotein-related genes [67]. The downregulation of this gene within the context of this study may either indicate a deficiency in SELO among birds impacted with Wooden Breast or that seloprotein concentrations are locally diminished within breast muscle of affected birds. In contrast to the downregulation of *SELO*, another family of antioxidative enzymes glutathione peroxidase 7 and 8 (*GPX7*, *GPX8*) are observed to be upregulated in birds affected with Wooden Breast, potentially because of elevated ROS levels and also to compensate for changes to SELO. In past studies, GPX7 has been shown to provide antioxidant functions against high levels of ROS in order to combat oxidative damages in humans [68]. It was also suggested that the presence of GPX7 is needed in order to maintain low ROS levels in specific cell types like oesophageal cells found in the human throat [68]. Another gene that is upregulated in affected birds and likely serves an anti-oxidative function is carbonic anhydrase 3 (*CA3*). Within the current study, *CA3* is one of the top differentially expressed genes with a fold change of 28.47. It has been proposed that CA3 has the ability to serve as an anti-oxidizing agent against damaging molecules, has both regulatory and reparative functions, and is a critical player in the antioxidant defense system within skeletal muscle [45].

### Fiber-type switching

In the current study, one of the most differentially expressed genes in affected samples was myoglobin (*MB*). Myoglobin is mainly distributed within Type I or aerobic muscle (slow twitch) fibers and serves to store oxygen. High myoglobin content is typically correlated with “red” meat, which has a high density of Type I fibers [69]. However, the pectoralis, a “white” muscle in chickens, consisting almost entirely of type II, or anaerobic (fast twitch) fibers, and in which myoglobin has previously either not been detectable [70] or observed at very low levels [71, 72]. In fact, the lack of myoglobin and increased glycogen content in Type II fibers account for the white color of pectoral meat [69]. Therefore high expression of myoglobin in the pectoralis muscle of broilers is unexpected and may possibly be explained by the phenomenon fiber-type switching, or a change from fast twitch to slow twitch fibers in response to myofiber degeneration and necrosis observed in the Wooden Breast myopathy. Muscle fibers are extremely dynamic and tend to adapt to environmental cues [73]. The phenotypic composition of muscle fibers can be altered by various stressors such as activity rate, mechanical stress, hormones, aging, response to muscle damage, and/or changes in muscle oxygenation [73]. These events have the capacity to modify the functional properties of a given muscle fiber, possibly leading to fiber-type switching [73].

Fiber type switching from fast-to-slow twitch fibers has also been suggested in turkeys exhibiting PSE muscle conditions [74]. Upregulation of troponin I type I, skeletal slow, (*TNNI1)* in the present study may support the hypothesis of fast-to-slow switch in Wooden Breast myopathy. *TNNI1* expression likewise differs by muscle type and, similar to *MB*, is also limited to expression in only slow skeletal fibers [75]. The *TNNI* subfamily has further been found to play a role in the mediation of skeletal muscle relaxation and contraction [76]. Other slow skeletal fiber-type isoforms upregulated in this study include myozenin 2 (*MYOZ2)* and myosin binding protein C, slow type (*MYBPC1). MYOZ2* expression has been reported in only cardiac and slow-twitch skeletal muscle, and acts to control for calcineurin, which is a positive regulator of slow-type fibers [77, 78]. Similarly, MyBPC1 is one of three MyBPC isoforms, and interacts with slow skeletal muscle to regulate muscle contraction [79]. In this study *TNNI1*, *MYOZ2*, and *MyBPC1* are upregulated in affected birds, potentially indicating fiber-type switching to type I fibers following myofiber damage in the Wooden Breast myopathy.

Jordan et al. 2004 [80] suggest that ryanodine receptor 1 (RyR1) in the pectoralis muscle in birds may act to suppress slow muscle specific genes. This is supported by high levels of reactive oxygen species (ROS) or nitric oxide (NO), which can irreversibly impact RyR1 subunits as a consequence of oxidation [81]. Consequently, the inhibition of RyR1 by ryanodine may lead to a fast-to-slow fiber type switch [80]. Mutation to the *RyR1* gene in pigs has been previously correlated with the occurrence of malignant hyperthermia, a skeletal muscle myopathy, which leads to defects in Ca^2+^ channel functioning [82], PSE-type meat [82], and rigidity of the muscle caused by hypermetabolism [83]. It has also been suggested that inositol trisphosphate receptor 1 [84], protein kinase C alpha and theta [85] and muscarinic acetylcholine receptor (M1) [86], possess critical roles in fiber-type switching, although in the current study these genes remain insignificant. Fiber-type switching has also been characterized in the callipyge sheep muscle condition. This condition in sheep involves muscles within the pelvic region that exhibit an increase in muscle mass as well as leaner muscle and is associated with an increase in feed efficiency [87]. Callipyge sheep show some similar symptoms to birds affected with Wooden Breast such as hypertrophy of the affected muscle and localization to specific muscle regions; however, it is suggested that these sheep actually undergo a slow-to-fast fiber-type switch [88]. Thus, though there is indication that fiber-type switching may be occurring during Wooden Breast in chickens, the pathways altered for fiber-type switching are likely different in broiler chickens than those exhibited in myopathies of other species, such as the callipyge phenotype in sheep.

### Cellular repair

Many genes involved in cellular repair and transport are differentially expressed within our dataset. It is highly probable that these genes are linked to the Wooden Breast myopathy in an effort to compensate for muscle damage in this degenerative muscle disease.

One of the top genes upregulated in affected chickens is cysteine and glycine-rich protein 3 (*CSRP3*) (fold change = 23.17), which is also referred to as muscle LIM (Lin11, Isl-1, and Mec-3) protein within the literature [89, 90]. *CSRP3* expression is present only in striated muscle, including skeletal muscle, and it plays a large role in myogenic differentiation [91]. The expression of *CSRP3* also correlates with myotube growth and formation [91], which is a key feature of muscle repair. Barash et al. 2005 [92] studied *CSRP3* knockout mice and found that deficient mice displayed an array of skeletal muscle defects such as shorter sarcomere length and muscle atrophy. They suggested that CSRP3 has a role in the maintenance of skeletal muscle and also supports skeletal muscle regeneration following injury through structural repair and gene regulation [92]. *CSRP3* may significantly increase in expression after periods of exercise in humans [93]. This may occur in order to promote muscle growth as CSRP3 has also been linked to involvement in muscle hypertrophy and regeneration through the calcineurin/NFAT signaling pathway and myogenic differentiation (MYOD) expression [91]. It is also interesting to note that *CSRP3* is mainly expressed in slow skeletal muscle, compared to its low expression in fast skeletal muscle [94]. Similar to this, *CSRP3* expression was upregulated during fast-to-slow fiber type switch in rats [94]. Therefore, as discussed above, fiber-type switching may be occurring in birds with Wooden Breast as a secondary response to the disease, acting as a mechanism of myofiber repair and/or regeneration under an altered redox homeostasis. Xu et al. 2009 [90] also suggest that the expression of *CSRP3* could impact meat quality since CSRP3 is involved in both myofiber generation and fiber-type switching.

Along a similar line, another highly upregulated gene in this study is nephroblastoma overexpressed (*NOV*) also referred to as *CCN3* [95] (fold change = 7.33). NOV is a member of the CCN family of proteins, which contains cysteine-rich angiogenic protein 61 (CCN1), connective tissue growth factor (CCN2), and nephroblastoma overexpressed (CCN3) [96]. This protein family is an important role player for many biological functions such as cell migration, proliferation, differentiation, and extracellular modification [96]. It is also thought that the *CCN* gene family takes part in critical roles during cell development, wound healing, tissue homeostasis, and varying conditions including cancer and fibrosis [95]. Heath et al. 2008 [95] conclude from their results that *NOV* is necessary and required for normal muscle maintenance and use, showing that the over-expression of *NOV* leads to increased cell proliferation and promotes the survival of cells expressing myogenin. Myogenin is an essential regulator of myogenesis, and crucial to muscle maintenance, regeneration, and repair [97], and is overexpressed in musculoskeletal tumors [98]. *NOV* expression has also been previously linked with multiple components of cellular repair, as well as wound healing and endothelial adhesion and migration [99]. It is likely that NOV is involved in the Wooden Breast disease acting as a regulator of cellular and tissue repair through myoregeneration and cellular movement.

Other differentially expressed genes within the current study, such as musculoskeletal embryonic nuclear protein 1 (*MUSTN1*) also contribute to cellular repair and regeneration in skeletal muscle. In the current study *MUSTN1*, which is considered essential for skeletal muscle growth and regeneration [93, 100], is upregulated in affected birds. Increased expression of *MUSTN1* has also been reported in domestic chickens; its expression is higher in broiler chickens than it is in layer chickens. This observation supports the notion that *MUSTN1* may be involved in the regulation of muscle hypertrophy [101]. *MUSTN1* expression was also upregulated in correlation to exercise-induced muscle damage, supporting its possible involvement in muscle damage repair [93]. As both affected and unaffected birds in the present study were from the same genetic lineage of broiler chickens, upregulation of *MUSTN1* in affected birds likely indicates compensatory hypertrophy or muscle repair secondary to muscle damage rather than physiologic hypertrophy during bird growth.

Other key contributors to the actin cytoskeleton system, such as members of the Rho GTPase family, could also indicate cell differentiation or movement in the reparative phase of the disease. Within our dataset there are four members of this family that are upregulated. These include: Rho GTPase activating protein 10 (*ARHGAP10*), Rho GTPase activating protein 20 (*ARHGAP20*), and Rho GTPase activating protein 40 (*ARHGAP40*). Another significant member of this family that is downregulated is Rho-related BTB domain containing 3 (*RHOBTB3*). The Rho GTPase family are important regulators of the organization of the actin cytoskeleton [102]. Rho activation is necessary to maintain strong cellular and focal adhesion between cells [102], and Rho-GTPase proteins are involved in many general functions such as cell migration, cell proliferation, and cytoskeleton reorganization [103]. As an example, it is thought that the upregulation of *ARHGAP10* is essential to cell differentiation within the cytoskeleton [104]. Although some of the functions of Rho GTPase genes remain unknown, it is possible that the expression of these genes within the current study is due to disruption within the actin cytoskeleton and subsequent repair from the damage manifested by the Wooden Breast myopathy.

Results from IPA show that the canonical pathways “Actin Cytoskeleton Signaling” (p-value = 3.18E-07) and “Axonal Guidance Signaling” (p-value = 1.27E-06) have probable functions in tissue repair during Wooden Breast muscle disease. The “Actin Cytoskeleton Signaling” pathway contains 36 significant differentially expressed genes from our gene list. Most of the genes within this pathway relate to specific growth factors such as, epidermal growth factor (*EGF*) and fibroblast growth factor (*FGF*), and also include many cytoskeleton related genes such as, actin related protein complex (*ARPC*) and many member of the myosin heavy and light chain families (*MYH*/*MYL*). As shown in Fig. 1 the major endpoints of this pathway deal with the assembly, polymerization, and the stabilization of both actin and myosin. We hypothesize that the “Actin Cytoskeleton Signaling” pathway is mainly dealing with secondary response to injury in the form of cellular repair in affected muscle. It is important to point out the growth factors associated with this pathway include epidermal growth factor (*EGF*) and fibroblast growth factor 10 (*FGF10*) which are upregulated, and fibroblast growth factor 1 (*FGF1*) which is downregulated. It is more likely that growth factors are expressed differently in affected birds due to repair and regeneration occurring in the diseased muscle. The upregulation of *EGF* is important since *EGF* is previously known to promote myoblast proliferation to aid in muscle repair [105]. However, the downregulation of *FGF1* is unexpected, since it is known that FGF1 contributes greatly to skeletal muscle proliferation [106]. Mitchell et al. 1999 [107] also found a downregulation of *FGF1* when studying the response to muscle stretch, or stretch hypertrophy, in chickens. Mitchell et al. 1999 [107] were inconclusive as to why this phenomenon was occurring, but hypothesized that stretch itself may contribute to downregulation of FGF1. Also seven members of the myosin family are differentially expressed within the “Actin Cytoskeleton Signaling” pathway including three myosin heavy chain genes (*MYH2, MYH6, MYH10*), and four myosin light chain genes (*MYL1, MYL9, MYL12B, MYLK2*). Myosin is essential for muscle contraction and comprises most of the myofibrillar proteins within muscle cells [108]. Within this pathway myosin is functioning to create “actomyosin assembly contraction” (Fig. 1, IPA®). The assembly and contraction of the actomyosin complex is essential as both actin and myosin are responsible for muscle contraction [109].

The other canonical pathway related to cellular repair presented by IPA is “Axonal Guidance Signaling” (Additional file 1). This pathway contains 56 significant differentially expressed genes from our gene list. Some of the gene families within this pathway include the A Disintegrin and Metalloproteinase (ADAM) metallopeptidase family, myosin light chain family, and the SEMA (Semaphorins) domain family. Since the muscle and surrounding connective tissues are undergoing injury, inflammation, and edema in Wooden Breast, it is possible that there is a simultaneous disruption and repair in axons of nerve fibers within the muscle. As reported through IPA some of the possible functions of this pathway include, cytoskeleton reorganization, actin filament reorganization, axon outgrowth, and axon guidance. Several genes within this pathway are regulators of cellular repair and regeneration in the muscle and axon systems. For example, *ADAM12*, which is upregulated in affected birds, is most often expressed in adult muscle during regeneration, and may take part in reducing necrosis and inflammation [47]. Secondly, *SEMA3A*, which is upregulated in affected birds, acts to promote intramuscular re-innervation in damaged muscle fibers in order to restore muscle contraction [110]. It is also interesting to note that, clinically, birds with Wooden Breast typically display a drastic decrease in the amount of muscle fasciculations after death compared to normal birds (data not shown). Although this phenomenon remains to be studied more fully, it is possible that damage to either muscle or nerve fibers have led to loss of function within the muscle, though there is no significant histologic evidence of primary nerve damage within affected muscle. From the results given through IPA we postulate that both the “Actin Cytoskeleton Signaling” and “Axonal Guidance Signaling” canonical pathways are not likely primary causes of the Wooden Breast myopathy, but are secondary to the disease, differentially expressed during the repair phase of the illness.

### Coagulation system pathway and related genes

**Fig. 2:**
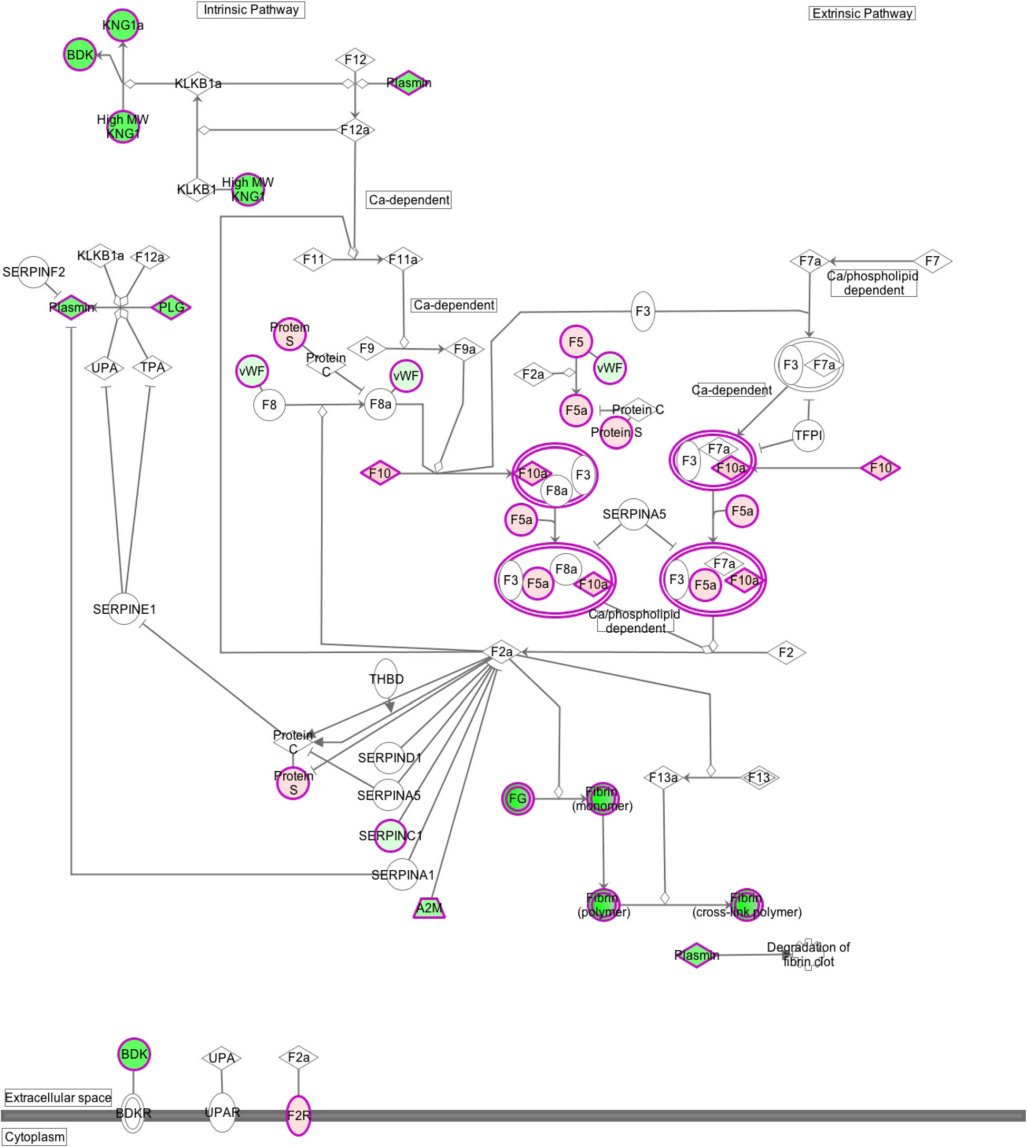
The “Coagulation System” pathway highlighting differentially expressed genes in birds affected with Wooden Breast myopathy. Canonical pathways were identified and depicted by Ingenuity Pathway Analysis (IPA®) software. This pathway shows the “Coagulation System” as related to significant DE genes in affected birds from the sample population. Genes that are highlighted in green are considered to be downregulated in affected birds. Genes that are highlighted in red are considered to be upregulated in affected birds

**Fig. 3:**
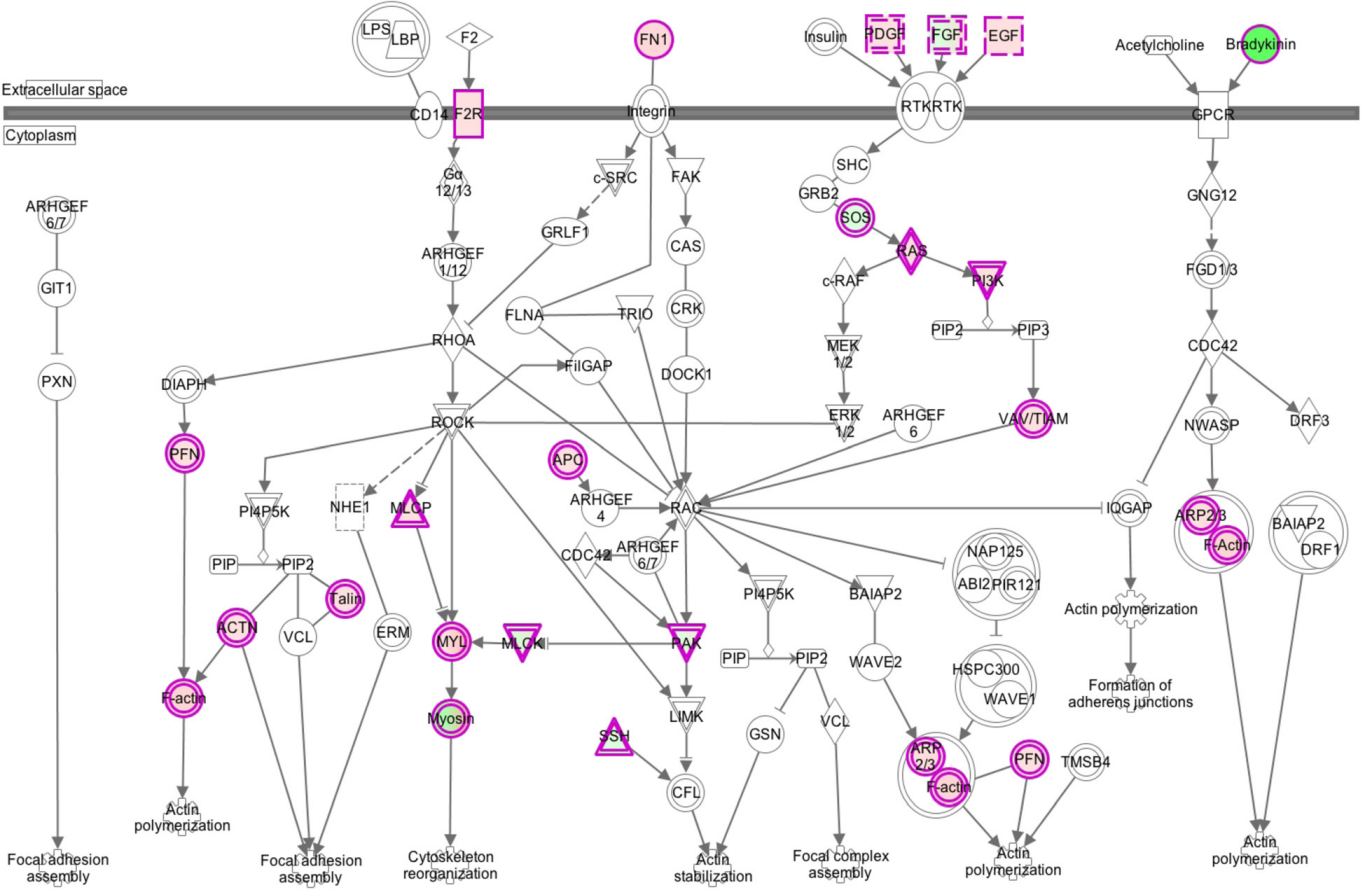
The “Actin Cytoskeleton Signaling” pathway highlighting differentially expressed genes of birds affected with Wooden Breast myopathy. Canonical pathways were identified and depicted by Ingenuity Pathway Analysis (IPA®) software. This pathway shows the “Actin Cytoskeleton Signaling” as related to significant differentially expressed genes in affected birds from the sample population. Genes that are highlighted in green are considered to be downregulated in affected birds. Genes that are highlighted in red are considered to be upregulated in affected birds

The inflammation observed microscopically in birds affected with Wooden Breast suggests that genes involved in the coagulation system pathway would likely be upregulated due to vascular (venous) damage and inflammation. It is highly regarded that inflammation promotes the activation of coagulation [111]. However, our data supports the downregulation of genes within the coagulation system pathway in affected brids as given by IPA® (Fig. 2). It should be noted that the genes involved in the coagulation system pathway are mainly expressed in the liver, where most of the coagulation factors are synthesized, rather than in muscle. The DE genes involved within the coagulation pathway identified by the present study include: alpha-2-macroglobulin (*A2M)*, fibrinogen alpha chain *(FGA),* fibrinogen beta chain *(FGB),* and fibrinogen gamma chain (*FGG)*, kininogen 1 (*KNG1)*, plasminogen (*PLG)*, serpin peptidase inhibitor, clade A, member 1 (*SERPINC1)*, and von Willebrand factor (*VWF),* which are all found to be downregulated (Fig. 3). Other DE genes within this pathway which are upregulated include: coagulation factor V (*F5)*, coagulation factor X (*F10)*, coagulation factor II (*F2R)*, and protein S (alpha) (*PROS1)*. The reasons for the involvement of the coagulation pathway and its overall downregulation in Wooden Breast remain to be elucidated.

### Biological significance of gene expression profile

Features of the gene expression profile (increase in intracellular calcium, hypoxia, and oxidative stress, etc.) brought to light by this study have strong correlation to the histologic lesions reported previously by Sihvo et al. 2014 [6] and observed in diagnostic specimens evaluated by our laboratory (data not shown).

A major characteristic of increased intracellular calcium levels is myofiber hypercontraction. Hypercontraction associated with skeletal muscle degeneration has been previously identified across species [112, 113]. It is suggested that the accumulation of Ca^2+^ within the cell leads to hypercontraction either as a result of poor Ca^2+^ -ATPase performance [114] or because of interference with sarcolemma integrity [115]. Ultimately hypercontraction results in cell degeneration and necrosis [114]. Histologically hypercontraction can be established through the observation of irregular Z bands or as hyaline fibers, which form as a result of excess calcium [116, 117] and leads to the coagulation of the contractile proteins within the cells [117]. Although irregular Z bands have not been a prominent feature of Wooden Breast cases, swelling and hyalinization of fibers is the predominant myofiber lesion present in affected Delmarva poultry (data not shown) and has also been reported by Bilgili 2013 [8]. The “hypereosinophilic” nature of myofibers described by Sihvo et al. 2014 [6] would also correlate to hyaline degeneration of myofibers. Furthermore, it is highly likely that the microscopic swelling and coagulation of proteins observed to be widespread within affected muscle fibers may be contributing to clinically and grossly evident hardening of the pectoral muscle as a whole. These observations support the hypothesis of hypercontraction and/or contractile protein denaturation, potentially due to increased levels of intracellular calcium. It should also be noted that no significant mineralization within the muscle was observed grossly or microscopically to suggest dystrophic calcium deposition in degenerating or necrotic myofibers. As such, it appears that calcium overload, as supported by gene expression profiling results, mainly affects the contraction state of myofibers in Wooden Breast.

Birds affected with Wooden Breast also show characteristics of histologic changes and clinical symptoms associated with hypoxia [6, 8], potentially due to vascular damage or inadequacy [8]. From previous histologic reports [6, 8] it is unclear whether poor vasculature is the inciting cause of hypoxia or if there are other contributing factors, which establish a hypoxic environment. Hypoxia and oxidative stress have been continually linked in research, as in the present study. As mentioned, from RNA-seq results, it is likely that the pectoralis major muscle undergoes hypoxic events in Wooden Breast. Although vascular damage is present in affected birds, this is only seen on the venous side, rather than the arterial side of the vascular system; as such, there is little microscopic evidence to suggest impairment of oxygenated blood delivery to the tissues unless there is a subtle difference in vascular density between affected and unaffected birds. However, venous damage, supported by histologic evidence of the accumulation of immune cells surrounding and within veins in affected muscle, as reported by Sihvo et al., 2014 [6] and as observed in our laboratory (data not shown), could ultimately impair the removal of cellular metabolic waste products, leading to the build-up of oxidative metabolites, and triggering further damage to surrounding cells. Inflammation in the muscle may also be contributing to or responding to a hypoxic state in the tissue.

To study the impact of hypoxic environment in mice, Lopes-Ferreira et al. 2001 [48] investigated skeletal muscle degeneration using fish venom and reported histological outcomes associated with the hypoxic environment including, inflammation, infiltration of macrophages and leucocytes, fiber necrosis, variations in fiber size, hypercontracted fibers, as well as fibrosis. These microscopic findings are similar to those in Wooden Breast. Fibrosis in particular is a common sequela of muscle repair under hypoxic conditions, as the occurrence of fibrosis aims to replace lost muscle tissue with collagen and fibrocytes [48]. Fibrosis is evident microscopically in Wooden Breast muscle, and several genes within the collagen family have been determined to be upregulated in the present study, including: *COL3A1*, *COL4A3*, *COL4A4*, *COL4A5*, *COL5A1*, *COL5A2*, *COL6A1*, *COL6A2*, *COL6A3*, *COL8A1*, *COL8A2*, *COL11A1*, *COL12A1*, *COL14A1*, *COL16A1*, *COL21A1*, *COL24A1*, *COL25A1*, and *COL27A1*. Furthermore, there is additional microscopic evidence to corroborate a hypoxic state; beyond fibrosis, the accumulation of lipid within affected muscle is also apparent histologically as variably-sized discrete vacuoles within the sarcoplasm of affected muscles (data not shown). Hypoxia has been previously linked with increases in lipid storage in cultured cells [118], cardiac muscle [119], and also in macrophages [120]. In support of the previous studies [6, 8], the present study revealed various differentially-expressed genes between affected and unaffected birds that may be correlated with a hypoxic state within the pectoralis major muscle of chickens with Wooden Breast. Hypoxia appears to be a critical factor in the Wooden Breast myopathy, though it is not apparent whether it is a primary cause for muscle lesions or is occurring secondary to inflammation and myofiber swelling from another cause.

Previous muscle disorders described in chickens, such as deep pectoral myopathy (DPM), have been associated with hypoxia resulting from restricted blood flow to the muscle as a predominant cause of muscle degeneration [1, 121]. The incidence rate of DPM is typically associated with high-yield breast muscle birds with high growth rate [1]. In the authors’ experience, Wooden Breast also has a high correlation with the growth rate and breast muscle weight of commercial broilers; most cases occur in breeding lines associated with fast growth, high feed efficiency, and high breast muscle yield. However, unlike in DPM, necrosis is limited to individual myofibers rather than widespread, and the superficial pectoral muscle is more affected than the deep pectoral muscle. Regardless, as Sihvo et al. 2014 [6] mention, it is highly likely that the high growth rate and increased breast muscle weight of modern-day broilers are key factors in myodegenerative disorders, including Wooden Breast and deep pectoral myopathy.

The gene expression profile brought to light within this study and previously reported histology of Wooden Breast suggests a pro-inflammatory response is taking place in affected birds, which involves the influx of immune cells. In the human muscular disease, Duchenne muscular dystrophy (DMD), cellular membrane damage caused by high intracellular calcium levels and inflammation leads to immune responses observed by a dramatic rise in inflammatory cell concentration within the affected area [122, 123]. Immune cells are known to generate ROS during inflammatory events in order to stimulate phagocytosis [124]; in mammals, ROS is formed by neutrophils and macrophages through NADPH-oxidase, a membrane-bound molecule [125]. ROS produced by immune cells has the ability to cause direct damage to muscle tissue or may act indirectly through the activation of catabolic signaling through immune molecule interaction with muscle receptors [126]. Histologic lesions for Wooden Breast suggest signs of oxidative stress because of observations such as fiber degeneration, variation in myofiber size, membrane and cellular rupture, inflammation, immune cell response, and replacement of myofibers with connective tissue and/or fat [6, 8]. Sihvo et al. 2014 [6] suggest that broiler selection for large breast muscle size coupled with an increased growth rate may cause broilers to be more vulnerable to oxidative stress.

Histologically, it is seen that tissue repair is occurring through mechanisms such as inflammation, myofiber regeneration, and fibrosis [6], as well as possibly through fiber-type switching. Canonical pathways reported by IPA such as “Actin Cytoskeleton Signaling” and “Axonal Guidance Signaling” also support attempted regeneration and repair of the muscle. The infiltration of immune cells to the area may support these efforts to repair damaged tissues. Macrophages are essential for removing damaged muscle proteins to allow regenerative fiber replacement; some macrophages have pro-regeneration qualities helping with muscle regeneration associated with their function [127]. Fibrosis and myofiber regeneration, both of which are also supported by the differentially expressed genes in our study, are other examples of microscopic observations that support a reparative phase is taking place. Ultimately, gene expression profiling undertaken in the present study has strong correlation to known microscopic lesions present in Wooden Breast.

## Conclusion

Using RNA-sequencing and pathway analysis tools such as IPA, we were able to define possible primary and secondary factors affecting the breast muscle of affected chickens. We were further able to link RNA-sequencing data to histologic findings in our lab and previously published reports of Wooden Breast myopathy [6] to show potential biologic significance for the characteristic gene expression profile observed in Wooden Breast. Through the analyses of differentially expressed genes, significant differences were observed in genes associated with intracellular calcium, possible fiber-type switching, hypoxia, and oxidative stress (Table 3). Although it remains difficult to characterized if features of the disease are primary or secondary, it is likely that all play a significant role in the pathogenesis of this disease. Also, we were able to establish a trend of significant genes and pathways related to cellular repair, which are likely occurring secondary to tissue damage in the Wooden Breast myopathy.

**Table 3:** Summary of main categories of genes involved in Wooden Breast myopathy separated by category

Gene category	Gene name	Symbol	RNA-seq fold change	Nanostring fold change
Fiber-Type Switching:				
	Myoglobin	MB	14.3	12.4
	Myosin Binding Protein C, slow type	MYBPC1	9.1	16.2
	Myozenin 2	MYOZ2	7.8	7.7
	Troponin I type I, skeletal slow	TNNI1	4.0	N/A
Intracellular Calcium:				
	ATPase, Ca++ transporting, cardiac muscle, slow twitch 2	ATP2A2	2.1	N/A
	Cytosolic phospholipase A2	PLA2G4A	2.3	N/A
	Parvalbumin	PVALB	8.1	7.2
	Phospholipase B1	PLB1	3.4	N/A
Hypoxia:				
	6-phophofructo-2-kinase	PFKFB3	−4.1	N/A
	Arylhydrocarbon Receptor Nuclear Translocator 2	ARNT2	2.2	1.7
	Asparagine synthetase	ASNS	4.4	N/A
	Matrix metallopeptidase 2	MMP2	2.2	N/A
	Muscle-specific carbonic anhydrase III	CA3	28.5	25.5
	Procollagen-lysine, 2-oxoglutarate 5-dioxygenase 2	PLOD2	2.5	N/A
	Transforming growth factor beta 3	TGFB3	3.9	3.7
	Transient receptor potential cation channel, subfamily A	TRPA1	6.7	N/A
Oxidative Stress:				
	Corticotrophin-releasing hormone	CRH	14.3	13.3
	FK506 binding protein	FKBP51	−1.9	N/A
	Glutathione peroxidase 7	GPX7	2.3	N/A
	Glutathione peroxidase 8	GPX8	2.1	N/A
	Heat shock 27 kDa protein family, member 7	HSPB7	3.2	4.3
	Heat shock 70 kDa protein 4-like protein	HSPA4L	1.5	N/A
	Heat shock 105 kDa/110 kDa protein 1	HSPH1	2.2	N/A
	Heat shock transcription factor 2	HSF2	2.9	N/A
	Muscle-specific carbonic anhydrase III	CA3	28.5	25.5
	Selenoprotein O	SELO	−2.1	N/A
Cellular Repair:				
	ADAM metallopeptidase domain 12	ADAM12	2.2	N/A
	Cysteine and glycine-rich protein 3	CSRP3	23.2	27.3
	Epidermal growth factor	EGF	1.6	N/A
	Fibroblast growth factor 1	FGF1	−2.1	−1.9
	Fibroblast growth factor10	FGF10	2.4	1.3
	Musculoskeletal embryonic nuclear protein 1	MUSTN1	4.9	6.0
	Myosin, heavy chain 13, skeletal muscle	MYH13	−4.7	1.0
	Myosin, light chain 2, skeletal slow	MYL3	5.4	1.5
	Myosin, light chain 12A regulatory	MYL12A	1.9	N/A
	Myosin light chain kinase 2	MYLK2	−2.6	N/A
	Nephroblastoma Overexpressed	NOV	7.3	3.0
	Rho GTPase activating protein 10	ARHGAP10	2.2	N/A
	Rho GTPase activating protein 20	ARHGAP20	2.4	N/A
	Rho GTPase activating protein 40	ARHGAP40	3.0	2.8
	Rho-related BTB domain containing 3	RHOBTB3	−11.9	−5.1
	Sema domain, Ig, short basic domain, secreted, 3A	SEMA3A	1.6	N/A

For each gene category, the complete gene name, symbol, fold change from RNA-seq data, and fold change from Nanostring® data are provided. Positive and negative fold change values support respectively upregulation and downregulation of gene expression in birds affected by Wooden Breast myopathy relative to the gene expression in unaffected birds

The present work should be considered a first step towards elucidating the underlying genetic fingerprint of the Wooden Breast myopathy in commercial broilers. With the RNA-sequencing profile identified by this work, there is a potential to find specific genetic markers characteristic for this disease for use in diagnosis and detection of subclinical or carrier birds. Future work with RNA-sequencing across the time course of disease development would be beneficial. Since birds in the present study were all collected at market age, performing a time-series study using RNA-sequencing could help determine the specific age of onset of this disease and may help distinguish primary from secondary mechanisms contributing to Wooden Breast and associated gross and histologic lesions. Histochemical staining for specific fiber-type could further be implemented to confirm if a fiber-type switch is occurring in affected birds. Since it is also possible that a single gene or a few candidate genes are principle causal factors in this disorder, conducting a genome-wide association study (GWAS) could also be greatly beneficial.

To our knowledge, this is the first study on the Wooden Breast disease in commercial broiler chickens to focus on RNA-sequencing data and gene expression profiling. Although the underlying cause of this disease still remains unknown, this work has brought forward many factors associated with the pathogenesis of this muscle disease. This work further shows the importance of using RNA-sequencing in biologic correlation with gross and microscopic disease lesions to study and compare muscle disorders across species. While more research must be performed to gain insight into the specific origin of Wooden Breast myopathy in chickens, RNA-sequence profiling has the potential to directly contribute to disease diagnosis and research for myopathies across animal species, including humans.

### Availability of supporting data

Samples used in this study were obtained from Heritage Breeders, LLC. The data supporting the results are included within the article and its additional files. Readers may contact the corresponding author for additional information.

## Additional files

Additional file 1:
**Sample identity verification.**
Additional file 2:
**Total number of reads per sample.**
