# Supplementary material for: Characterization of a novel chicken muscle disorder through differential gene expression and pathway analysis using RNA-sequencing
Source: BMC Genomics. 2015 May 21;16(1):399. doi: 10.1186/s12864-015-1623-0 (PMC4438523; doi:10.1186/s12864-015-1623-0)
Supplement: Additional file 1: — Sample identity verification. [file 12864_2015_1623_MOESM1_ESM.docx]

**Additional file 1.**

*Sample identity verification*

To verify the identity of the samples used in our RNA-seq experiment, we applied both genotyping of genomic DNA (gDNA) with high-density SNP arrays and SNP genotype calling from the RNA-seq data. For genotyping with SNP arrays, gDNA was isolated from blood samples by QIAGEN® DNeasy Blood and Tissue Kit following the manufacturer’s protocol for nucleated blood. The gDNA samples were genotyped using the high-density 600k chicken SNP array from Affymetrix ® [1] in which 580,961 SNPs were included.

For SNP calling from the RNA-seq data, we applied a 2-step variant calling method to reduce reference allele bias. First, reads from RNA-seq of all samples were initially mapped to the latest assembly of the chicken reference genome (Galgal4.0) with 2-pass alignment in STAR [2], and then we followed the workflow of GATK for variant calling [3]. To reduce false positive calls, we eliminated the low quality variants (with read depth (DP) < 100, quality by depth (QD) < 2 or strand bias (FS) >30) and clusters with more than 2 SNPs within a window of 15 bp. By applying these criteria, we obtained 701,067 SNPs and INDELs (insertions and deletions) in the first step of variant calling. Secondly, we created a masked chicken genome in which all variant positions from the first step were masked to N for reads alignment, and then followed the same workflow of variant calling by GATK [3] and the same criteria for quality control. To further reduce reference allele bias, we excluded INDELs and the SNPs in less than 100bp from nearby INDELs because the reads carrying INDELs have less chance to be mapped to the reference genome [4]. After all, we obtained 201,079 SNPs on exonic regions of the chicken genome, of which 14,887 were overlapped with the SNPs from the 600k SNP array based on their physical positions. Of these overlapped SNPs, we only used homozygous loci for the identity verification of the samples used in our RNA-seq experiment, as heterozygous SNPs may express in allele specific ways. For each chicken, we matched its 600k SNP genotypes of homozygous loci with those in variant calling from RNA-seq data and used the percentage of matching genotypes for the identity verification of the samples.

We found the percentages of matching genotypes between SNP arrays and RNA-seq from the same birds were higher than 99.2 but between different birds, the percentages ranged from 58.4% to 68.5%. This information allowed us to verify the identity of all samples used in our RNA-seq experiment (see below Table 1).

**Table 1.** Matrix of percentages (%) of matching genotypes between 600K SNP array and RNA-seq

| SeqID* | Samples genotyped by 600k SNP chips | | | | | | | | | | |
| --- | --- | --- | --- | --- | --- | --- | --- | --- | --- | --- | --- |
|  | C51 | C52 | C53 | C55 | C56 | C57 | C59 | C62 | C63 | C64 | C66 |
| C51 | 99.4 | 59.7 | 59.5 | 60.0 | 61.4 | 59 | 58.9 | 59.7 | 60.7 | 60.5 | 60.3 |
| C52 | 62.8 | 99.4 | 63.6 | 62.4 | 62.6 | 64.5 | 64.3 | 64.3 | 64.8 | 65.4 | 63.8 |
| C53 | 61.0 | 62.1 | 99.4 | 61.2 | 62.9 | 61.5 | 63.5 | 63.6 | 62.8 | 64.0 | 60.9 |
| C55 | 60.8 | 60.3 | 60.5 | 99.2 | 62.6 | 66.4 | 62.4 | 63.4 | 62.7 | 60.1 | 62.0 |
| C56 | 64.7 | 63.1 | 64.7 | 64.9 | 99.3 | 62.4 | 65.5 | 65.5 | 63.4 | 62.7 | 65.1 |
| C57 | 60.9 | 63.2 | 61.9 | 67.6 | 60.8 | 99.4 | 61.5 | 63.2 | 65.0 | 62.5 | 63.7 |
| C59 | 59.3 | 61.5 | 62.2 | 61.9 | 62.5 | 60.1 | 99.4 | 63.6 | 62.6 | 65.1 | 61.2 |
| C62 | 61.1 | 62.4 | 63.3 | 63.7 | 63.3 | 62.7 | 64.5 | 99.5 | 63.2 | 63.2 | 68.5 |
| C63 | 60.7 | 61.7 | 61.3 | 61.6 | 59.8 | 63.1 | 62.2 | 61.7 | 99.5 | 62.4 | 60.7 |
| C64_1 | 60.9 | 62.2 | 63.2 | 63.6 | 63.1 | 62.5 | 64.5 | 99.5 | 62.9 | 63.1 | 68.3 |
| C64_2 | 61.7 | 63.4 | 63.5 | 60.4 | 60.8 | 61.8 | 66.0 | 63.1 | 63.7 | 99.5 | 61.7 |
| C66 | 59.5 | 59.6 | 58.4 | 60.1 | 60.4 | 60.6 | 59.9 | 65.9 | 59.7 | 59.5 | 99.4 |

Note: *: ID from RNA-seq samples. C64_1 stands for sample C64 in our first batch of cDNA library preparation for RNA-seq, which shows that its genotypes from RNA-seq are matched to sample C62’s genotypes on SNP array, marked red color in the table. Thus, we repeated the RNA isolation, cDNA library preparation and RNA-seq for C64 labeled as C64_2 in the table. Its percentage of matching genotypes between 600K SNP array and RNA-seq is 99.5%, marked green color in the table. Since NanoString data for this sample was obtained using C64_1 RNA sample, we excluded this sample from log2 (fold-change) estimation from Nanostring data.

References:

1. Kranis A, Gheyas AA, Boschiero C, Turner F, Yu L, Smith S, Talbot R, Pirani A, Brew F, Kaiser P, Hocking PM, Fife M, Salmon N, Fulton J, Strom TM, Haberer G, Weigend S, Preisinger R, Gholami M, Qanbari S, Simianer H, Watson KA, Woolliams JA, Burt DW: **Development of a high density 600K SNP genotyping array for chicken.** *BMC Genomics* 2013, **14**:59.

2. Dobin A, Davis CA, Schlesinger F, Drenkow J, Zaleski C, Jha S, Batut P, Chaisson M, Gingeras TR: **STAR: ultrafast universal RNA-seq aligner.** *Bioinformatics* 2013, **29**:15–21.

3. McKenna A, Hanna M, Banks E, Sivachenko A, Cibulskis K, Kernytsky A, Garimella K, Altshuler D, Gabriel S, Daly M, DePristo MA: **The Genome Analysis Toolkit: a MapReduce framework for analyzing next-generation DNA sequencing data.** *Genome Res* 2010, **20**:1297–303.

4. Stevenson KR, Coolon JD, Wittkopp PJ: **Sources of bias in measures of allele-specific expression derived from RNA-sequence data aligned to a single reference genome.** *BMC Genomics* 2013, **14**:536.
