# Supplementary material for: Characterization of a novel chicken muscle disorder through differential gene expression and pathway analysis using RNA-sequencing
Source: BMC Genomics. 2015 May 21;16(1):399. doi: 10.1186/s12864-015-1623-0 (PMC4438523; doi:10.1186/s12864-015-1623-0)
Supplement: Additional file 2: — Total number of reads per sample. [file 12864_2015_1623_MOESM2_ESM.docx]

**Additional file 2.**

Total number of reads per sample.

Total number of reads per sample for all (11) samples with an average of 97,890,272 reads per sample.

Summary of the differentially expressed genes in the “Axonal Guidance Signaling” Pathway from IPA®.

| Symbol | Entrez Gene Name | Fold Change |
| --- | --- | --- |
| ABLIM2 | actin binding LIM protein family, member 2 | 1.534 |
| ACTR3 | ARP3 actin-related protein 3 homolog (yeast) | 1.591 |
| ADAM8 | ADAM metallopeptidase domain 8 | 2.651 |
| ADAM11 | ADAM metallopeptidase domain 11 | -2.568 |
| ADAM12 | ADAM metallopeptidase domain 12 | 2.214 |
| ADAMTS1 | ADAM metallopeptidase with thrombospondin type 1 motif, 1 | -1.514 |
| ADAMTS2 | ADAM metallopeptidase with thrombospondin type 1 motif, 2 | 1.516 |
| ADAMTS6 | ADAM metallopeptidase with thrombospondin type 1 motif, 6 | 2.557 |
| ADAMTS7 | ADAM metallopeptidase with thrombospondin type 1 motif, 7 | 1.720 |
| ARPC5 | actin related protein 2/3 complex, subunit 5, 16kDa | 1.592 |
| ARPC1A | actin related protein 2/3 complex, subunit 1A, 41kDa | 2.597 |
| BMP7 | bone morphogenetic protein 7 | 1.994 |
| C9orf3 | chromosome 9 open reading frame 3 | 2.153 |
| COPS5 | COP9 signalosome subunit 5 | -1.653 |
| CXCR4 | chemokine (C-X-C motif) receptor 4 | 1.973 |
| EGF | epidermal growth factor | 1.594 |
| EPHA3 | EPH receptor A3 | 2.422 |
| EPHB3 | EPH receptor B3 | 1.801 |
| FZD1 | frizzled class receptor 1 | 1.990 |
| GNA14 | guanine nucleotide binding protein (G protein), alpha 14 | -5.738 |
| GNG2 | guanine nucleotide binding protein (G protein), gamma 2 | 1.850 |
| HHIP | hedgehog interacting protein | -2.551 |
| HRAS | Harvey rat sarcoma viral oncogene homolog | 1.823 |
| LINGO1 | leucine rich repeat and Ig domain containing 1 | -3.306 |
| LRRC4C | leucine rich repeat containing 4C | -5.124 |
| MET | MET proto-oncogene, receptor tyrosine kinase | 1.815 |
| MMP2 | matrix metallopeptidase 2 (gelatinase A, 72kDa gelatinase, 72kDa type IV collagenase) | 2.183 |
| MMP9 | matrix metallopeptidase 9 (gelatinase B, 92kDa gelatinase, 92kDa type IV collagenase) | 2.337 |
| MMP11 | matrix metallopeptidase 11 (stromelysin 3) | 2.545 |
| MMP13 | matrix metallopeptidase 13 (collagenase 3) | 2.461 |
| MRAS | muscle RAS oncogene homolog | 2.989 |
| MYL3 | myosin, light chain 3, alkali; ventricular, skeletal, slow | 5.358 |
| MYL9 | myosin, light chain 9, regulatory | 1.732 |
| MYL10 | myosin, light chain 10, regulatory | 4.976 |
| MYL12B | myosin, light chain 12B, regulatory | 1.941 |
| PAK1 | p21 protein (Cdc42/Rac)-activated kinase 1 | 1.687 |
| PAK3 | p21 protein (Cdc42/Rac)-activated kinase 3 | -1.504 |
| PDGFD | platelet derived growth factor D | 1.949 |
| PFN2 | profilin 2 | 2.077 |
| PIK3R6 | phosphoinositide-3-kinase, regulatory subunit 6 | 1.688 |
| PLXNA2 | plexin A2 | -1.590 |
| PLXNC1 | plexin C1 | 1.585 |
| PRKCH | protein kinase C, eta | -2.143 |
| ROBO1 | roundabout, axon guidance receptor, homolog 1 (Drosophila) | 2.144 |
| ROBO3 | roundabout, axon guidance receptor, homolog 3 (Drosophila) | 4.484 |
| SEMA3A | sema domain, immunoglobulin domain (Ig), short basic domain, secreted, (semaphorin) 3A | 1.560 |
| SEMA3G | sema domain, immunoglobulin domain (Ig), short basic domain, secreted, (semaphorin) 3G | -1.874 |
| SEMA7A | semaphorin 7A, GPI membrane anchor (John Milton Hagen blood group) | -2.359 |
| SLIT3 | slit homolog 3 (Drosophila) | 3.103 |
| SOS1 | son of sevenless homolog 1 (Drosophila) | -1.655 |
| TUBA8 | tubulin, alpha 8 | -2.487 |
| TUBA1B | tubulin, alpha 1b | 2.399 |
| TUBA1C | tubulin, alpha 1c | -1.714 |
| TUBB6 | tubulin, beta 6 class V | 1.791 |
| WNT11 | wingless-type MMTV integration site family, member 11 | 2.511 |
| WNT2B | wingless-type MMTV integration site family, member 2B | 2.380 |

Summary of significant DE genes their respective fold change expressed in affected birds in found within the “Axonal Guidance Signaling” canonical pathway. This table is a product of Ingenuity Pathway Analysis (IPA®).
